# The incidence and mortality rates due to stroke and myocardial infarction following implementing the package of essential non-communicable diseases; A historical cohort study

**DOI:** 10.34172/jcvtr.2022.32

**Published:** 2022-09-07

**Authors:** Sayed Ali Ayat, Shayeste Rostami, Reza Khadivi

**Affiliations:** Community Medicine Department, Medical Faculty, Isfahan University of Medical Sciences, Isfahan, Iran

**Keywords:** Cardiovascular Diseases, Incidence, Mortality, Myocardial Infarction, Stroke

## Abstract

***Introduction:*** The package of essential non-communicable diseases (PEN) has been implemented in 2016 in Iran. The present study aimed to evaluate the incidence rate of myocardial infarction (MI) and stroke, and the mortality rate due to these diseases, following the lunch of the PEN project.

***Methods:*** This is a historical cohort study that was performed in 2020. Data were gathered through the hospital information system in the exposed and the control counties hospitals in the Isfahan province. The data about over 30-year-old patients who were hospitalized as definite cases of MI and/or stroke were collected via census sampling. The incidence rates of MI, stroke, and mortality due to those diseases were compared in 2015 (one year before the launch of the PEN project) and 2019 (4 years after the project initiation).

***Results:*** Following the implementation of the PEN project, the incidence and mortality rates due to MI among the over 30-year-old population in the exposed county, were reduced by 0.31 per 1000 and 9 per 100 000 respectively. Furthermore, the incidence rate due to stroke in the exposed county further slowed down, and the mortality rate due to stroke was reduced by 33 per 100,000 more than 30 years old population significantly.

***Conclusion:*** Following the implementation of the PEN project, the incidence rate due to MI and mortality rates due to MI and stroke decreased significantly. In addition, the incidence rate due to stroke further slowed down in the exposed county in comparison with the control county.

## Introduction

 Cardiovascular diseases (CVD) are the main cause of death and disability-adjusted life years (DALY’s) in adults around the world. It is estimated that 80% of the burden of CVDs occurs in developing countries.^1^

 The main risk factors related to CVD that are remarkable and modifiable include Hypertension (HTN), Diabetes Mellitus (DM), dyslipidemia, smoking, alcohol consumption, central obesity, and physical inactivity.^2^

 The World Health Organization (WHO) has wished for a 25% reduction of death from non-communicable diseases (NCD) for those who are above 30 by 2025, compared to the mortality rate in 2010. In Iran, 82.2% of mortality, following NCDs, occurs from cardiovascular diseases, cancers, chronic pulmonary diseases, and diabetes.^3^ On the other hand, controlling these risk factors through training and lifestyle modification, early diagnosis, and early treatment can reduce the incidence of cardiovascular events such as MI and Stroke, hospitalization rate, costs, and mortality associated with CVDs.^4,5^

 The Health Sector Evolution Plan (HSEP) was carried out in 2014 in the Iranian health system. It mainly focused on Universal Health Coverage (UHC), and about 95% of Iranian citizens were covered by governmental health insurance. Besides, co-payments for inpatient services were reduced in public hospitals to less than 6% for citizens in large and medium cities and less than 3% for inhabitants in small cities and rural regions.^6^

 In conjunction with HSEP and to achieve the WHO’s goals of reducing the NCD burdens, the Package of Essential Non-communicable diseases (PEN) was implemented as a pilot project in the Shahreza county in Isfahan Province (in the center of Iran) and 3 others counties in different provinces in 2016 according to WHO PEN guideline. To attain PEN’s goals, the interventional package was conducted to prevent and control four principles, chronic non-communicable diseases including HTN, DM, hyperlipidemia, and chronic obstructive pulmonary disease, and their common risk factors such as lack of activity, unhealthy diet, smoking, and substance abuse.

 To attain the reduction of the 10-year risk of cardiovascular events in the PEN project, people over 30-years of age undergo interventions such as screening, 10-year cardiovascular events risk assessment, training for lifestyle modification, early treatment, and/or referring to a specialist as needed. Those duties are delivered in all comprehensive health centers either rural or urban regions in pilot counties by active manner and free of charge. The wage of general practitioners in the outpatient health care system changed from salaries to per capita.^7^ This study aimed to evaluate the incidence rate of myocardial infarction (MI) and stroke, and the mortality rate due to these diseases following the lunch of the PEN project in two distinct counties in Isfahan Province, one as the intervention county for piloting the PEN project and the other as the control county, in comparison with before that.

## Material and Methods

###  Study Design/ Extraction methods

 This historical cohort study was performed from May-1-2020 to September-15- 2020. The PEN project was implemented in the Shahreza county in Isfahan Province in February 2016. For the present study, two distinct counties in Isfahan Province have been selected: the Shahreza county as the exposed county and the Najafabad county as the control county. The exposed county is similar to the control county in terms of socio-economic and cultural patterns. In each county, there were two exclusive hospitals.

 The data about all patients over 30 years old who were admitted with a definite diagnosis of MI or/and stroke and/or died in hospitals due to those diseases in 2015 (one year before launching the PEN project) and in 2019 (4 years after the program was launched) were entered in this study.

###  Data sources/ Data Collection

 After coordination with the authorities of Isfahan University of Medical Sciences (MUI) and officials of teammate hospitals, and approval of the ethics committee in MUI, the data were collected through census sampling according to hospital information system (HIS) and other non-electronic files in two exclusive hospitals in the intervention county and two exclusive hospitals in the control county from April 2015 to April 2020.

###  Inclusion and exclusion criteria

 All of these hospitals had at least one internal medicine specialist, one cardiologist, and one neurologist over the years of the present study. According to the International Statistical Classification of Diseases and Related Health Problems 10^th^ revision (ICD-10), each patient above 30-years of age who was hospitalized in the exposed and the control counties hospitals in 2015 and 2019 with a definite diagnosis of acute MI (I21.0-I21.9) and/or stroke (I64) and/or died in hospitals due to those diseases, was included in the present study as a new case regardless of the type of gender or residence.^8,9^ The temporary hospitalized patients (those with less than 6 hours of hospitalization) and patients hospitalized with a diagnosis other than MI such as unstable angina, any neurological disease other than strokes such as a transient ischemic attack, and all psychiatric disorders were excluded.

 Data related to the population of the over 30 in each county, were obtained from the statistics unit in the provincial health center.

###  Statistical analysis

 The data collected were coded by the year and name of the county and analyzed by SPSS statistics ver. 16.0 (SPSS Inc., Chicago, IL. USA) software, using descriptive statistics tests (frequency, relative frequency, mean and standard deviation) and analytical statistical tests (Chi-square, Independent T-test). *P *value < 0.05 was considered significant.

 The indicators about the admission rate (as the incidence rate), and the mortality rate due to MI and/or stroke were compared in over 30-year-old populations by different genders in each county (exposed and control) in 2015 and 2019.

## Results

###  Demographic characteristics

 The number of the over 30-year-old population and the mean and standard deviation of their ages in two different years (2015 vs. 2019) were presented in Table 1. As shown below, the number over 30-year-old population in the control county was two times more than in the exposed county, but the mean age in the exposed county was more than that in the control county.

**Table 1:** The number of over 30-year-old population and the mean and standard deviation of the age of inhabitants who stayed in the exposed and the control counties in 2015 and 2019.

**The Type of County**	**2015**		**2019**	
	**The number of over 30-year- old population**	**The mean±SD of age (Year) **	**The number of over 30-year-old population**	**The mean±SD of age (Year)**
The exposed	71 352	46.56 ± 15.8	85 413	47.6 ± 18.7
The control	164 093	45.2 ± 13.2	191 076	46.7 ± 13.7

###  The incidence rates of MI and Stroke

 Upon implementing the PEN project, the incidence rate of definite MI cases in the exposed county was different from to control county significantly (*P *value = 0.014, 95% CI: -1.382 – -0.205). The difference in difference (DID) showed a reduction as likely as -0.31 in the incidence rate of MI in 1000 over 30-year-old population.

**Figure 1:**
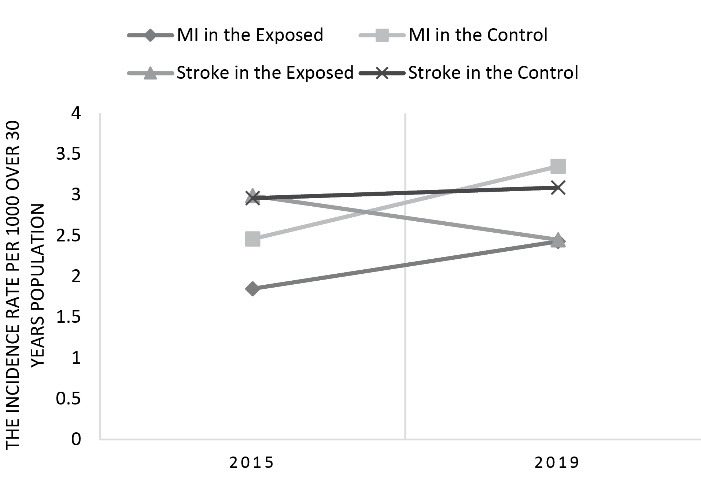
The incidence rate of MI and Stroke in the exposed and the control counties (per 1000 over 30 years population) in 2015 and 2019

 While the incidence rate of stroke in the exposed county revealed a reducing scheme, however, the incidence rate of stroke in the exposed county in comparison to the control county wasn’t different significantly (*P* value = 0.087). The DID showed a reduction as likely as -0.67 in the incidence rate of stroke in 1000 over 30-year-old population. The relative risks (RRs) of MI and stroke in the exposed county compared to the control county, showed a decreasing pattern in 2019 (RR < 1). (Table 2, Figure 1).

**Table 2:** The incidence rate of definite Myocardial Infarction and Stroke cases in the exposed and the control counties (per 1000 over 30 years population) in 2015 and 2019

**Type of cardio-vascular events**	**The type of county**	**2015**			**2019**			***P*** value*
		**Number**	**Incidence rate**	**Relative risk**	**Number**	**Incidence rate**	**Relative risk**	
Myocardial infarction	The exposed	132	1.85	0.75	208	2.43	0.72	0.014
	The control	404	2.46		641	3.35		
Stroke	The exposed	214	2.99	1.01	210	2.45	0.79	0.087
	The control	487	2.96		591	3.09		

*Independent T-test

 As seen in Table 3, there was no difference between the incidence of MI and stroke, based on gender, between the exposed and control counties (*P* value = 0.189 and *P* value = 0.062 respectively).

**Table 3:** The incidence rate of Myocardial Infarction and Stroke by gender (per 1000 over 30-year-old population) in the exposed and the control counties in 2015 and 2019

**Cardiovascular events**	**The type of county**	**Sex**	**2015**	**2019**	***P*** value*
Myocardial Infarction	The exposed	Male	1.47	1.83	0.189
		Female	0.38	0.6	
	The control	Male	1.63	2.14	
		Female	0.83	1.21	
Stroke	The exposed	Male	1.70	1.27	0.062
		Female	1.29	1.18	
	The control	Male	1.46	1.46	
		Female	1.50	1.63	

⁎Chi square, Fisher exact test

###  The mortality rate due to MI and Stroke in hospitalized patients

 Data analysis revealed a significant difference in the intrahospital mortality rate in MI patients between the exposed and control counties hospitals after launching the PEN project (*P *value = 0.029, 95%CI: -16.22 – -10.14). The DID value in mortality rate due to MI between the exposed and the control counties were -9 cases per 100,000 population.

**Figure 2:**
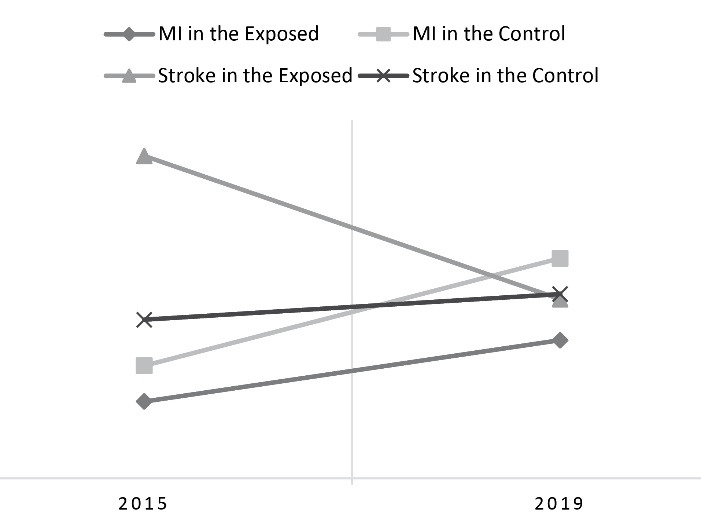
The Mortality rate due to MI and Stroke in the exposed and the control counties (per 100 000 over 30 years population) in 2015 and 2019

 The intrahospital mortality rate due to stroke in the exposed county hospitals reduced significantly too (*P *value = 0.019, 95% CI: 16.55 – 22.08). The DID value in mortality rate due to stroke between exposed and control counties revealed that following implementing the PEN, 33 deaths per 100,000 population were prevented. In addition, the RRs of mortality rate due to MI and stroke in the exposed county compared to those in the control county showed a decreasing schema in 2019 (Relative Risk < 1). (Table 4, Figure 2).

**Table 4:** The intrahospital mortality rate due to Myocardial Infarction and Stroke (per 100 000 over 30-year-old population) in the exposed and the control counties in 2015 and 2019

**Cardiovascular events**	**The type of county**	**2015**		**2019**		***P*** value*
		**Mortality rate**	**Relative risk**	**Mortality rate**	**Relative risk**	
Myocardial Infarction	The exposed	15	0.682	27	0.628	0.029
	The control	22		43		
Stroke	The exposed	63	2.032	35	0.972	0.019
	The control	31		36		

*Independent T-test

## Discussion

 Following the implementation of the PEN project,the incidence and mortality rates due to MI among the over 30-year-old population in the exposed county were reduced by 0.31 per 1000 and 9 per 100 000 respectively. Furthermore, the incidence rate due to stroke in the exposed county further slowed down, and the mortality rate due to stroke was reduced by 33 per 100,000 more than 30 years old population significantly.

 A long time–series studies that were done in developed countries showed that following policies that focus on control of risk factors for cardiovascular disease such as HTN, DM, blood cholesterol concentration, and cigarette smoking, through program implementation objecting community empowerment to change lifestyle, cessation cigarette smoking, consume fresh fruit and vegetables, reducing fat dairy products, and improve utilization of effective drugs in lowering blood pressure and blood cholesterol, they lead to ether reduce in the prevalence of HTN and blood cholesterol nearly 50% or decreasing the risk of premature death in last decade in 20th century. In contrast to developed countries, the prevalence of cardiovascular diseases’ risk factors such as HTN, DM, blood cholesterol concentration, and cigarette smoking have been raised in developing countries in past decades.^10-12^ Developing countries are facing main challenges such as diversity in social determinants of health issues, complexity in health needs, and change in disease burden. Policy-makers in developing countries to achieve final goals including improving the health status, financial risk protection, and customer satisfaction, are accentuating the health sector reform.^11^

 To achieve those final goals, one of the fundamental approaches is facilitating the utilization of essential health services by at-risk populations. Since uninsured patients have utilized less frequent disease screening, particularly blood pressure screening, they suffered a higher mortality rate than patients who have had health insurance coverage.^13^ Recent studies that were performed after health sector reform implementation as UHC in Iran, revealed that the hospitalization rate has raised in comparison with the time before that.^14^ This way, the main obstacle facing poor people was removed and high-risk patients could be screened or cured easily. In this way, the non-receiving services patients in the present study would be insignificant.

 Our findings are similar to the study that was conducted on WHO-PEN in Bhutan. The findings showed that the implementation of the PEN program in the primary healthcare setting led to improving HTN and DM control.^15^

 Also, the study of Hyon et al, in the Democratic People’s Republic of Korea (North Korea) showed a significant relationship between the PEN project and reducing the risk of cardiovascular events, which is consistent with the current study.^16^

 Moreover, implementing prevention strategies to control risk factors can greatly reduce the incidence of intracerebral hemorrhage and improve the prognosis of the survivors.^17^

 The results of the study by Zhang et al. indicate that the implementation of WHO PEN can have a significant effect on reducing the incidence of MI and stroke, with controlling HTN as the main risk factor too.^18^

 Those studies reported the results of the WHO PEN in the early phases in which community-based prevention and control of cardiovascular events in Bhutan, North Korea, and China were focused on resident screening, general population awareness, treatment, and control of HTN. In such a way, they emphasized attending primary health care physicians in training sessions and enhancing the physician skills for practicing in WHO PEN guidelines. They haven’t reported measurable outcomes such as the incidence rate of either MI or stroke or premature death due to cardiovascular accidents yet. It seems between developing countries that launched the WHO PEN in their primary health care delivery systems, the present study is the first study that reports the main project’s objectives of reducing the rate of MI and stroke and the mortality rate due to those risks factors in the general population.

 In the present study, the DID value revealed that after launching the PEN project, it has happened a reduction in new MI and stroke cases by 0.31 and 0.67 per 1000 over 30 years old population respectively. Based on the study by Law et al, that was performed on 147 clinical trials, revealed that a decrease in blood pressure by drug treatment can decrease the rate of MI by 22% and stroke by 41%.^19^ Their participants in those studies included over 57-year-old population, who were more susceptible to CHD events or stroke, while the PEN’s target population were over 30-year-old persons. On the other hand, this study was conducted only 4 years after the implementation of the PEN interventions. However, it seems it required more time for an accurate evaluation of PEN measurements.

 In the present study, no difference was found in the incidence of MI and stroke by gender between the exposed and control counties. It seems that following the expansion of government health insurance coverage for uninsured people and improving access to essential health services on the one hand and changing the payment system for health providers to capitation on the other hand, and increasing the responsiveness of health providers to health care consumers, the utilization of essential health services such as screening of blood pressure, blood cholesterol, blood glucose, and in continuity change in lifestyle was improved in both sexes.^20^

 One of the strengths of this study is the large sample size in two distinct counties the exposed and control counties. However, the main limitation of the present study is the short duration between the implementation of the PEN project and the assessment of the outcomes: At the time of the present study, only 5 years had passed since the start of the PEN project. it seems that the evaluation of the PEN project in reducing the incidence and mortality rates of MI and/or stroke should be followed in the coming years. In addition, in recent years, following the national integrated non-communicable diseases control package in the health delivery system, the rest comprehensive health centers in the Isfahan province carried out the Ira-PEN project gradually. Thus, the contamination of over 30 years old people in the control county wasn’t inevitable and the efficiency of the PEN project would estimate lower than the real effects on the community.

 In the present study, we gathered the data from exclusive two hospitals in every exposed and control county based on HIS. This information system, there weren’t gathered the history of the years in which every residence stayed in the exposed or the control county. So, those residents with a short staying in either the exposed or the control county can be misleading in the project’s results. In addition, we considered every admission with a definite diagnosis of MI or stroke according to ICD-10 criteria as a new case and took into account the incidence of MI or stroke. Unfortunately, current HIS in-teammate hospital weren’t able to differentiate those admissions from re-admission. However, this phenomenon was similar in either exposed or control county, and in a long time about 5 years the duration of the study, it doesn’t seem to be a major confounder.

 Based on the patient triage system in hospitals affiliated with MUI, all patients with the emergency condition in each county must refer to the local hospital in their county, and in continuity, if they need, they would refer to subspeciality hospitals in the center of the province in Isfahan city. Thus, it seems that either those patients from the exposed or the control counties with similar emergency conditions who have been admitted to other hospitals in Isfahan province would be negligible. Besides, since the data gathering in the present study was carried out on census sampling in every two exclusive hospitals in exposed and the control county in either electronic or non-electronic files, it seems that the missing data would be insignificant.

## Conclusion

 Following the implementation of the PEN project in Iran, the incidence rate and the inpatient mortality rate due to myocardial infarction decreased in the exposed county, compared to the control county significantly. While the incidence rate due to stroke slowed down in the exposed county, it should be noted that the inpatient mortality rate due to stroke in the exposed county decreased significantly compared to the control county too.

## Acknowledgments

 We are thankful to Dr. Behrouz Keleidari, the Vice-Chancellor, Dr. Hamid Ganji, the director, and other respectful experts in Clinical Affairs and Shahreza and Najafabad hospitals and those health networks in Isfahan University of Medical Sciences for their invaluable help. We are also grateful to the Deputy of Research and Technology of Isfahan University of Medical Sciences for their research support.

## Funding

 This study was supported by the vice chancellor for Research and Technology of Isfahan University of Medical Sciences. This paper was taken from thesis No. 398166 in the Medical Faculty of Isfahan University of Medical Sciences. No fund, grant, or other support was received.

## Ethical approval

 The present study was performed according to the agreement of Isfahan University of Medical Sciences (MUI) ethics committee No. IR.MUI.MED.REC.1398.152.

## Competing interest

 The authors have no conflicts of interest associated with the material presented in this paper.
